# Three-dimensional dose uncertainty maps based on the fraction of field edge dose for volumetric modulated arc therapy plans

**DOI:** 10.1016/j.phro.2025.100802

**Published:** 2025-06-28

**Authors:** Emmanouil Terzidis, Fredrik Nordström, Magnus Gustafsson, Anna Karlsson, Julia Götstedt, Anna Bäck

**Affiliations:** a Department of Medical Radiation Sciences, Institute of Clinical Sciences, Sahlgrenska Academy, University of Gothenburg, Gothenburg, Sweden; b Department of Therapeutic Radiation Physics, Medical Physics and Biomedical Engineering, Sahlgrenska University Hospital, Gothenburg, Sweden

**Keywords:** Field edge, EAM, Absorbed dose uncertainty, Plan complexity, VMAT

## Abstract

**Background and purpose:**

Absorbed dose uncertainties in radiotherapy plans are generally larger near field edges compared to the center of the field. The aim of this study was to investigate dose uncertainties related to the field edge in 3D for plans of varying complexities.

**Materials and methods:**

A method was developed for calculation of the fraction of field edge dose (FED), that could be visualized as a 3D uncertainty map (3DUM_FED_). Twelve clinical treatment plans were included for four different treatment sites that were reoptimized to create one plan with reduced complexity and one of increased complexity. 3DUM_FED_ was calculated for all 36 plans. The highest FED for a 2 cm^3^ volume (FED_2 cm_^3^) and average FED (FED_mean_) were calculated for the planning target volumes (PTV) and organs at risk (OAR) and compared with the edge area metric (EAM).

**Results:**

High FED (above 20 %) were mainly found just outside the PTV border. FED_mean_ in PTV was highest for the plans of increased complexity. The FED_mean_ for PTVs and OARs, as well as the FED_2 cm_^3^ for PTVs, correlated with ρ ≥ 0.81 to EAM. The FED_2 cm_^3^ for OARs had a weaker correlation with EAM (ρ = 0.55). 3DUM_FED_ analysis revealed that plan complexity affects different parts of the patient volume in different ways.

**Conclusions:**

3DUM_FED_ offers a way to estimate dose uncertainties related to the field edge in 3D. It also allows for separate evaluation in different regions of interest, unlike EAM, which mainly correlates with the dose uncertainty related to the PTV.

## 1 Introduction

Modern advanced radiotherapy techniques, such as volumetric modulated arc therapy (VMAT), often include complex field apertures, i.e., smaller field sizes and more irregular apertures compared to conventional three-dimensional conformal radiotherapy (3DCRT) techniques [1]. Such complex beam apertures can lead to larger uncertainties, i.e., differences between calculated and delivered dose. The dose differences may arise from limitations in the dose calculation method within the treatment planning system (TPS), such as a suboptimal model of the radiation source or calculation limitations in regions with a lack of charged particle equilibrium [2]. Additionally, they can result from delivery variations in the treatment machine [3,4] e.g. an incorrectly positioned multi-leaf collimator (MLC) or jaw. Absorbed dose uncertainties due to the aforementioned factors manifest mainly in the penumbra region, i.e., the field edge region [2,5].

Complexity metrics have been suggested for estimating dose uncertainties in treatment plans based on the beam fluence map, the aperture shape of the beam and/or the degree of modulation of treatment machine parameters [6]. Only a few metrics have shown a correlation to deviations between measured and calculated dose. Complexity metrics that focus on the field edge in relation to the beam aperture have been found to correlate to differences between calculated and measured dose [[7], [8], [9]]. One example of such a metric is the Edge Area Metric (EAM), introduced by Götstedt et al [5,9]. EAM can be used to evaluate the complexity associated with aperture size and shape at control point (CP) level with values ranging from 0 to 1, where 1 corresponds to maximum complexity. EAM can be useful to estimate uncertainties in treatment plans before treatment delivery. However, it does not provide spatial information on where these uncertainties occur in the patient, making it challenging to assess their clinical impact. To date, there are no complexity metrics specifically designed to quantify plan complexity in relation to the patient geometry. Therefore, there is a need for a novel way to present complexity, one that quantifies dose uncertainty both numerically and spatially in 3D.

The aim of this work was to investigate absorbed dose uncertainties related to the field edge in 3D and thereby extend the concept of the EAM into the three dimensions of the patient volume.

## 2 Materials and methods

### 2.1 Method for calculating 3DUM_FED_

An in-house software, written in Visual C# (Microsoft Corporation, Redmond, WA, USA) was developed to calculate the fraction of absorbed dose originating from a field edge in 3D. The Eclipse scripting application programming interface (ESAPI) was used to interact with the Eclipse TPS (Varian Medical Systems, Inc., Palo Alto, CA, USA). The fully automatic software workflow comprises the following steps for a VMAT plan:

1. Each arc was divided into multiple static fields, one for each control point (CP) with the option to further divide into sub-CP levels. These static fields were defined by gantry, collimator, and couch angles, MLC and jaw positions, and monitor units (MU) in accordance with the arc’s specified parameters.
2. The dose distribution for each static field was calculated using a volume dose calculation algorithm available in Eclipse.
3. For each static field, the center coordinates of each voxel in the patient volume were projected onto the beam’s eye view (BEV) geometry at the source axis distance (Fig. 1). The coordinate transformation was achieved using a method similar to that developed by Sherouse [10], which accounts for the rotation of the collimator and gantry, as well as the translation and rotation of the couch. If the projected center of a voxel lied within or exactly at a predefined distance (defined by the user) from the field edge (both inside and outside of the field), the dose in that voxel from that static field was labeled as field edge dose. In this context, the field edge was defined by the MLC or the jaws, depending on which device was collimating the beam.

**Fig. 1:**
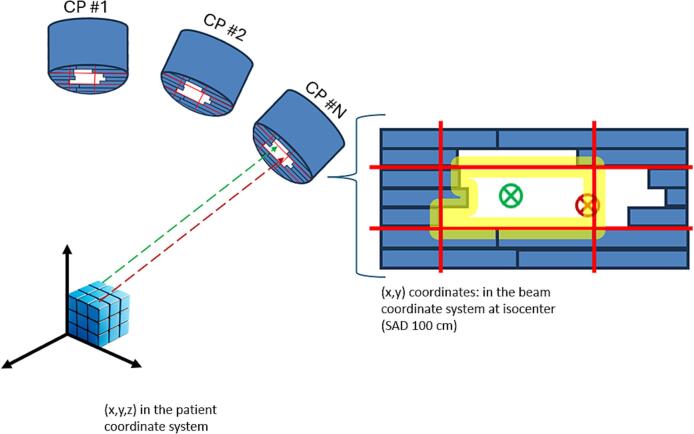
Illustration of voxel projection from the patient coordinate system to the beam coordinate system at isocenter (100 cm Source-to-Axis Distance, SAD) for an example control point (CP). The yellow region represents the area within a predefined distance from the field edge, as defined by the multi-leaf collimator (MLC, blue rectangles) and the jaws (solid red lines). The green dashed line and the green circle with an x represents a voxel projection where the voxel center is > a predefined distance from the field edge, i.e., outside of the field edge region. The red dashed line and the red circle with an x represents a voxel projection where the voxel center is within the yellow region, i.e., inside of the field edge region. (For interpretation of the references to color in this figure legend, the reader is referred to the web version of this article.)
4. For each voxel, the field edge dose from all static fields was summed. The total field edge dose was then divided by the total dose in that voxel to calculate the fraction of edge dose (FED), expressed as a percentage of the total dose. The resulting FED values created a 3D distribution across all voxels, which is referred to as the 3D uncertainty map (3DUM_FED_).

The 3DUM_FED_ could be overlaid on the patient’s CT scan and structures and could furthermore, be stored in Eclipse for further analysis using the tools available in the TPS.

### 2.2 Treatment plans

Twelve standard VMAT plans were included in this study, encompassing patients previously treated across various treatment sites: three for prostate cancer, three for head & neck cancer, three for lung cancer, and three for gynecological cancer. In head & neck cases, both the primary tumor site and lymph nodes were included, while prostate and lung cases involved only the primary tumor site. Gynecological cases varied: one had only a primary tumor site, another featured only a lymph node site, and the third included both primary tumor and lymph node sites. Originally, these plans were optimized using 6 MV photons (Photon Optimizer version 16.1.0) and calculated with the Analytical Anisotropic Algorithm (AAA version 16.1.0, 1.0 mm calculation grid) in Eclipse (Varian Medical Systems, Inc., Palo Alto, CA, USA). Treatment planning was conducted based on a TrueBeam linear accelerator equipped with Millennium MLCs (Varian Medical Systems, Inc., Palo Alto, CA, USA).

Each patient's clinical plan was reoptimized in the Eclipse TPS to create a simpler plan (i.e., less complex) and a more complex plan. The aim was to adjust the monitor unit (MU) objective and the level of the aperture shape controller (ASC) to achieve dose distributions similar to the clinical plan. Simple plans were generated with the highest ASC level, whereas ASC was deactivated for complex plans. The maximum MU limit constraint for simple plans was minimized without compromising target coverage, while the minimum MU limit constraint was raised for complex plans. Further details on MU constraints, prescribed doses and field parameters for each case are provided in the supplementary Tables S1-S4. The same dose objectives as used for the optimization of the clinical plan were initially used for the re-optimization, with minor adjustments if needed during the last optimization cycle. Additional information regarding the creation of simple and complex plans can be found in our previous work [4]. In total, 36 plans were considered in this study, i.e., three per case (clinical, simple and complex plans).

### 2.3 3DUM_FED_ analysis

The presented method to calculate 3DUM_FED_ (see section 2.1) was applied to each of the 36 VMAT plans. The AAA (version 16.1.0, 1.0 mm calculation grid) was used for the dose calculation of the static fields and the angular spacing of the CPs was 2 degrees. A distance of 2.5 mm inside and outside of the field edge was used to define the field edge region (Fig. 1). This distance was selected for a more direct comparison with the EAM complexity metric, which also was characterized by a 2.5 mm complex region around the field edge. In addition to 3DUM_FED_, the average FED (FED_mean_) was calculated separately for the planning target volume (PTV), and for specific organs at risk (OARs) where minimizing dose during plan optimization was a high priority. Specifically, FED_mean_ values were calculated for the rectum and bladder in prostate and gynecological cases, for the spinal cord, parotids and esophagus in head & neck cases, and for the spinal cord and lungs in lung cases. The near-maximum FED for a 2 cm^3^ volume (FED_2 cm_^3^, similar to the “near maximum dose” D_2 cm_^3^ in dose volume histogram analysis) was also calculated for the PTV and the aforementioned OARs.

### 2.4 Comparison with EAM

The average EAM over all CPs in each plan [5], was calculated for all 36 plans and was then compared to the corresponding FED_mean_ and FED_2 cm_^3^ for the PTV and for one OAR for each treatment site (rectum for prostate and gynecological cases, contralateral parotid for head & neck cases and spinal cord for lung cases). The relationships between FED_mean_ and FED_2 cm_^3^ with average EAM were assessed using Spearman's rank correlation coefficients (ρ).

## 3 Results

### 3.1 3DUM_FED_ analysis

**Fig. 2:**
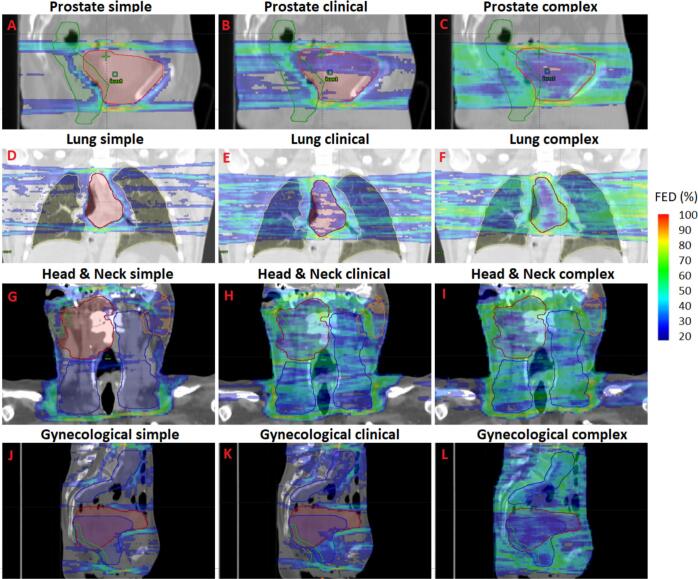
3D uncertainty maps based on the fraction of edge dose (FED). The magnitude of the FED is depicted with a color wash scale ranging from 20 % to 100 %. For each treatment site, the PTV-central slice is shown for plans of different complexity. Sagittal views are displayed for one prostate (A-C; case #1) and one gynecological case (J−L; case #3) while for the lung (D-F; case #3) and head & neck (G-I; case #2) cases coronal slices were chosen. The red contours indicate the primary PTVs, while the blue contours represent the lymph node PTVs. The dark green contours denote the rectum for prostate and gynecological cases. For the lung case, the lungs are outlined with yellow contour. For the head & neck case, the orange and green contours refer to the contralateral and ipsilateral parotids respectively. (For interpretation of the references to color in this figure legend, the reader is referred to the web version of this article.)

**Fig. 3:**
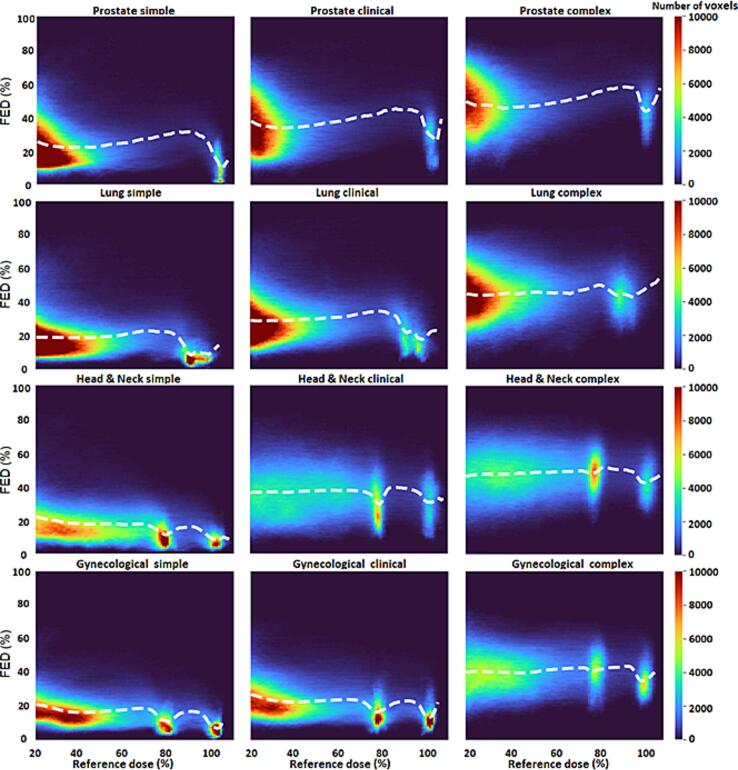
Two-dimensional histogram of the fraction of edge dose (FED) and the reference dose (i.e., calculated in the treatment planning system) in percentage of prescribed dose. Treatment plans are grouped separately for different treatment sites and complexity levels. The bin widths for the x- and y-axis are 2 % and 4 %, respectively. The white-dashed line represents the average value of FED for each x-bin (dose level). The color scale refers to the frequency of the voxels described by the same FED and dose value (i.e., frequency of voxels in each histogram pixel).

**Fig. 4:**
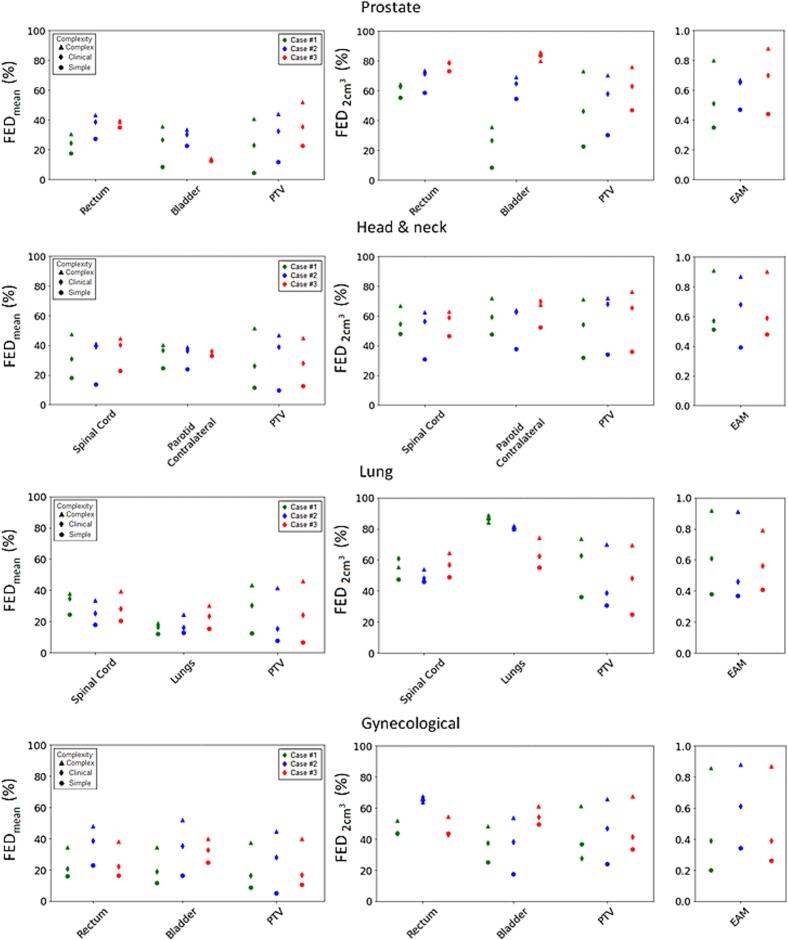
Average and near-maximum fraction of edge dose (FED_mean_ and FED_2 cm_^3^, respectively) for selected organs at risk (OAR) and the planning target volume (PTV), across all plans. FED_2 cm_^3^ refers to the highest value of FED received by a 2 cm^3^ volume. Edge area metric (EAM) scores are shown per plan for comparison. Each marker symbol represents a different complexity level and is color coded for different cases within the same treatment site (i.e. green refers to case #1, blue to case #2 and red to case #3). For cases with both a primary tumor volume and a lymph node target volume, the PTV refers to the sum of both PTV volumes. (For interpretation of the references to color in this figure legend, the reader is referred to the web version of this article.)

Computation time for a 3DUM_FED_ calculation (excluding the dose calculation time) in this work ranged from 7 s to 35 min. The highest FEDs were consistently observed just cranially and caudally to the PTV across all treatment plans (Fig. 2). For the simple plans, the vast majority of voxels with FED above 20 % were located outside the PTV, whereas for the clinical and complex plans, these voxels were observed also inside the PTV to a higher degree. Fig. 3 illustrates that the average FED for voxels within the same dose level generally increased across plans of increased complexity. The FED_mean_ for the PTV structure was always lowest for all the simple plans and highest for the complex plans (Fig. 4). A similar trend was observed for the OARs, with two exceptions: in prostate case #3, the highest FED_mean_ of the rectum was found in the clinical plan and in head & neck case #3, the highest FED_mean_ for the contralateral parotid was found in the clinical plan (Fig. 4).

### 3.2 Comparison with EAM

The EAM scores were consistently highest for the complex plans and lowest for the simple plans (Fig. 4). The FED_mean_ for the PTVs and OARs (i.e., rectum, spinal cord and contralateral parotid) as well as the FED_2 cm_^3^ for the PTVs were significantly (p < 0.05) correlated to EAM (ρ > 0.81). For OARs the FED_2 cm_^3^ was less correlated to EAM (Table 1). The 3DUM_FED_ analyses revealed that complexity affected different parts of the patient volume in different ways (Fig. 4). For instance, in head & neck case #3, EAM and FED_mean_ for PTV demonstrated a difference in complexity between simple, clinical and complex plans. However, the FED_mean_ for the contralateral parotid gland for the same case, showed little difference between the three complexity levels. A similar observation was made in lung case #1 and prostate case #3, regarding the lungs and bladder, respectively (Fig. 4). All values for FED_mean_, FED_2 cm_^3^, and EAM are provided in Supplementary Table S5.

**Table 1:** Spearman’s correlation coefficient (ρ) between the edge area metric (EAM) and the average fraction of edge dose and near maximum fraction of edge dose (FED_mean_ and FED_2 cm_^3^, respectively) for the planning target volume (PTV) and a selected organ at risk (OAR) (i.e., rectum for prostate and gynecological cases, contralateral parotid for the head & neck cases and spinal cord for the lung cases). Near maximum refers to the highest value of FED received by a 2 cm^3^ volume. The asterisk (*) indicates a statistically significant correlation (p < 0.05).

Correlation with EAM		
Structure	FED_mean_	FED_2 cm_^3^
PTV	ρ = 0.93*	ρ = 0.91*
OAR	ρ = 0.81*	ρ = 0.55*

## 4 Discussion

The proposed methodology to calculate 3DUM_FED_, was able to highlight anatomical regions with increased fraction of absorbed dose originating from a field edge region, thereby highlighting regions in the patient with increased dose uncertainty. Higher FED was observed in the regions outside but near the PTV compared to inside the PTV, indicating that OARs close to the target might be prone to increased dose uncertainty. FED was presented in 3D offering spatial information important for evaluating possible uncertainties in dose affecting target coverage or dose to OARs.

The computation time of the 3DUM_FED_ was reported excluding the dose calculation. Since the dose calculation is executed in the clinical TPS, the calculation time is dependent on the clinical setup. However, the 3DUM_FED_ calculation is performed concurrently with the dose calculation to reduce the overall computation time. The large variance observed in 3DUM_FED_ calculation times was case-dependent, primarily due to differences in dose calculation volumes and aperture shapes. The current implementation runs solely on a CPU and transitioning to GPU-based computation could decrease calculation time. Reduced calculation time would improve the method’s feasibility within a clinical workflow, potentially enabling the integration of 3DUM_FED_ information during treatment plan optimization.

Both 3DUM_FED_ and EAM focus on dose uncertainties related to the field edge region. FED_mean_ and FED_2 cm_^3^ for the PTV as well as the average EAM were consistently higher for plans with increased complexity and correlations between those FED values for the PTV and EAM were observed. However, this consistent pattern, i.e., higher values for plans with increased complexity, did not hold for the FED_2 cm_^3^ for OARs and for this parameter, a weaker correlation to EAM was observed. The stronger correlation between EAM and the FED values for the PTV indicates that EAM, though computed as an average for the entire plan without accounting for 3D geometry, has a stronger relation to the complexity in the PTV compared to the one in OARs. 3DUM_FED_ allows for evaluations of uncertainties separately in different regions of the irradiated volume and analyses revealed that issues related to the complexity of a plan can affect different regions within the patient volume differently. In some cases, even when EAM indicated a clear difference in plan complexity between three plans (i.e., simple, clinical and complex), the FED values for OARs did not necessarily follow the same trend. This means that, in some clinical situations where you aim to reduce complexity by reoptimizing the plan to reach a lower EAM, for example by using stricter ASC objective, the uncertainty due to the field edge might be reduced for the PTV but not necessarily at the same time for OARs. This highlights the gain and the clinical value of analyzing complexity in 3D rather than evaluating a composite complexity value for the whole plan. Just like EAM, most of the proposed complexity metrics for VMAT plans in the literature [6,11] presents a single value for the entire plan, which limits insight into where uncertainties manifest, and which anatomical regions are most affected. To our knowledge, no previous studies have reported on complexity evaluations in 3D. 3DUM_FED_ is an extension of EAM and comparisons between 3DUM_FED_ and EAM were performed in this work. EAM is an aperture-based complexity metric. Complexity metrics that relate to other aspects of complexity, e.g., dynamic parameters evaluated by metrics such as the Modulation index (MI) [12] and the Leaf sequence variability (LSV) included in the Modulation complexity score (MCS) [13], have been suggested. Previous comparisons between different types of complexity metrics have shown that there can be discrepancies in the evaluation of plan complexity, depending on the type of metric used [14,15]. Similar discrepancies are expected between 3DUM_FED_ and a 3D evaluation based on a different aspect of complexity. The concept of 3DUM can be used for 3D evaluations of any complexity aspect that can be quantified in a 2D BEV geometry. Although it is desired to reduce complexity and thereby dose uncertainties, there is a clinical trade-off between complexity and dose distribution optimality. Furthermore, a plan with an increased plan complexity resulting in increased FED values in the PTV might at the same time result in a dose distribution where an OAR is blocked and outside the field and the field edge region to a larger extent and thereby reducing the FED values in the OAR.

Complexity metrics are increasingly integrated in the field of radiotherapy, serving multiple roles. They have been suggested to be used during the plan optimization process to generate less complex plans or for comparing plans of similar dose distributions [7,[16], [17], [18]]. Another suggested application is to monitor how complexity of the produced plans in the clinic varies throughout the years, specifically after the introduction of new optimization algorithms [19]. Furthermore, complexity metrics can be used in quality audits or to evaluate how variations in treatment planning methodology across different institutions affect plan complexity [[20], [21], [22]]. 3DUM_FED_ provides information that could be relevant for each of these applications.

Similar to previously suggested plan complexity metrics [6,11], the 3DUM_FED_ calculations in this study presented absorbed dose uncertainty related to the field edge in a relative manner. While it is useful for comparing treatment plans, it does not provide the absolute absorbed dose uncertainty in a direct way. However, since the 3DUM_FED_ presents complexity evaluations voxel wise, further development of 3DUM_FED_ could enable the estimation of absolute absorbed dose uncertainty, enhancing its utility in evaluating plan quality. It should be noted, however, that 3DUM_FED_ only accounts for dose uncertainties related to the field edge, while other potential sources of uncertainty remain beyond the scope of this study.

The magnitude of the FED is dependent on the selection of the distance to field edge that defines the field edge region. A distance that is too high would give high FED values to a relatively larger number of voxels which can make it difficult to distinguish between voxels of reduced and increased dose uncertainty. On the other hand, using a low distance to field edge might underestimate the dose uncertainty. Therefore, additional work to determine the optimal distance to field edge would be valuable. This optimal distance might be specific for different treatment sites, i.e., different geometries, and for different beam energies. A 2.5 mm distance to the field edge was used to define the field edge region for EAM calculations because it was found to give the largest separation between lowest and highest EAM scores in a VMAT treatment plan [9]. In this work we used the same distance for the 3DUM_FED_ to enable a more direct comparison to EAM calculations.

The dose distribution for all treatment plans was calculated using the AAA. Since 3DUM_FED_ represents only a relative fraction of dose originating from the field edge, the influence of the dose calculation algorithm is small. However, 3DUM_FED_ calculations are more dependent on the choice of dose calculation grid size. In this study, a 1 mm grid size was used, which is the smallest available in the Eclipse TPS. A smaller grid size results in more voxels being projected in the BEV geometry, which in turn increases the spatial resolution of 3DUM_FED_.The accuracy of this method could be further improved by increasing the angular resolution when dividing the input VMAT arc into static fields.

In conclusion, 3DUM_FED_ is a novel method capable of estimating dose uncertainties associated with the field edge in VMAT plans in 3D. The highest fractions of field edge dose were typically observed in regions near and outside of the PTV border. This means that major concerns of dose uncertainties might relate to OARs close to the PTV, where the dose is often high relative to normal tissue tolerance. For plans of higher complexity, these high fractions of field edge dose also extended into the PTV. The evaluation of complexity in 3D using 3DUM_FED_ offers the possibility to separately evaluate dose uncertainties in different regions of interest within the patient compared to plan complexity metrics such as EAM that were found to mainly correlate to the dose uncertainty related to the PTV.

## Funding information

Varian Medical Systems, Inc., a Siemens Healthineers Company; King Gustaf V Jubilee Clinic Cancer Research Foundation.

## Credit authorship contribution statement

**Emmanouil Terzidis:** Conceptualization, Methodology, Validation, Formal analysis, Investigation, Data curation, Writing – original draft, Writing – review & editing, Visualization. **Fredrik Nordström:** Conceptualization, Methodology, Software, Validation, Investigation, Writing – review & editing, Supervision. **Magnus Gustafsson:** Conceptualization, Methodology, Investigation, Writing – review & editing. **Anna Karlsson:** Conceptualization, Methodology, Investigation, Writing – review & editing. **Julia Götstedt:** Conceptualization, Methodology, Investigation, Writing – review & editing. **Anna Bäck:** Conceptualization, Methodology, Investigation, Writing – review & editing, Supervision, Project administration, Funding acquisition.

## Declaration of competing interest

The authors declare the following financial interests/personal relationships which may be considered as potential competing interests: This work was partly financed by Varian Medical Systems, Inc., a Siemens Healthineers Company.
