# Supplementary material for: Three-dimensional dose uncertainty maps based on the fraction of field edge dose for volumetric modulated arc therapy plans
Source: Phys Imaging Radiat Oncol. 2025 Jun 28;35:100802. doi: 10.1016/j.phro.2025.100802 (PMC12271897; doi:10.1016/j.phro.2025.100802)
Supplement: Supplementary Data 1 [file mmc1.docx]

**Three-dimensional dose uncertainty maps based on the fraction of field edge dose for volumetric modulated arc therapy plans**

Emmanouil Terzidis^1,2^, Fredrik Nordström^1,2^, Magnus Gustafsson^2^, Anna Karlsson^1,2^, Julia Götstedt^1,2^, Anna Bäck^1,2^

^1^Department of Medical Radiation Sciences, Institute of Clinical Sciences, Sahlgrenska Academy, University of Gothenburg, Gothenburg, Sweden

^2^Department of Therapeutic Radiation Physics, Medical Physics and Biomedical Engineering, Sahlgrenska University Hospital, Gothenburg, Sweden

Correspondence: Emmanouil Terzidis, Department of Medical Radiation Sciences, Institute of Clinical Sciences, Sahlgrenska Academy, University of Gothenburg, Gothenburg, Sweden, Gula Stråket 2B, 413 46, Gothenburg, Sweden.
Email: [emmanouil.terzidis@gu.se](mailto:emmanouil.terzidis@gu.se)

Funding information: Varian Medical Systems, Inc., a Siemens Healthineers Company; King Gustaf V Jubilee Clinic Cancer Research Foundation.

**Supplementary material**

**Supplementary table S1**: Treatment plan information for all prostate cases. Treatment plans characterized as simple, and complex have been derived from the clinical plan for each case by altering the Monitor Unit (MU) optimization options and the Aperture Shape Controller (ASC) settings.

|  | Prescribed dose (Gy) | Number of fractions | | Arc geometry | Total MU | MU Optimization  Min/Max/Strength | ASC |
| --- | --- | --- | --- | --- | --- | --- | --- |
| Prostate 1 simple | 70 | 35 | 2 full | | 351.5 | 50/400/100 | Very High |
| Prostate 1 clinical |  |  |  |  | 434.1 | 50/550/75 | Very Low |
| Prostate 1 complex |  |  |  |  | 930.2 | 900/1300/100 | Off |
| Prostate 2 simple | 66 | 22 | 2 full | | 700.3 | 50/700/100 | Very High |
| Prostate 2 clinical |  |  |  |  | 1003.3 | 70/1000/75 | Very Low |
| Prostate 2 complex |  |  |  |  | 1527.6 | 1400/1800/100 | Off |
| Prostate 3 simple | 66 | 22 | 2 full | | 800.1 | 70/800/100 | Very High |
| Prostate 3 clinical |  |  |  |  | 1108.7 | 70/1000/75 | Very Low |
| Prostate 3 complex |  |  |  |  | 1882.2 | 1400/2000/100 | Off |

**Supplementary table S2**: Treatment plan information for all head & neck cases. Treatment plans characterized as simple, and complex have been derived from the clinical plan for each case by altering the Monitor Unit (MU) optimization options and the Aperture Shape Controller (ASC) settings.

|  | Prescribed dose (Gy) | Number of fractions | Arc geometry | | Total MU | MU Optimization  Min/Max/Strength | ASC | |
| --- | --- | --- | --- | --- | --- | --- | --- | --- |
| Head & neck 1 simple |  |  | |  | 352.3 | 50/35/100 | Very High |  |
| Head & neck 1 clinical | 68/52.1 | 34 | | 2 full | 495 | 0/600/50 | Very Low |  |
| Head & neck 1 complex |  |  | |  | 1058.8 | 1000/2000/100 | Off |  |
| Head & neck 2 simple |  |  | |  | 351.1 | 10/350/100 | Very High |  |
| Head & neck 2 clinical | 68/52.1 | 34 | | 2 full | 611.2 | 70/700/50 | Off |  |
| Head & neck 2 complex |  |  | |  | 1171.1 | 1000/2000/100 | Off |  |
| Head & neck 3 simple |  |  | |  | 421.3 | 10/420/100 | Very High |  |
| Head & neck 3 clinical | 68/52.1 | 34 | | 2 full | 601.2 | 0/600/50 | Very Low |  |
| Head & neck 3 complex |  |  | |  | 1111.4 | 1000/2000/100 | Off |  |

**Supplementary table S3**: Treatment plan information for all lung cases. Treatment plans characterized as simple, and complex have been derived from the clinical plan for each case by altering the Monitor Unit (MU) optimization options and the Aperture Shape Controller (ASC) settings.

|  | Prescribed dose (Gy) | Number of fractions | Arc geometry | Total MU | MU  Min/Max/Strength | ASC |
| --- | --- | --- | --- | --- | --- | --- |
| Lung 1 simple |  |  |  | 350.1 | 200/350/100 | Very High |
| Lung 1 clinical | 68 | 34 | 2 halves | 505.1 | 0/450/50 | Moderate |
| Lung 1 complex |  |  |  | 895.3 | 1000/1500/100 | Off |
| Lung 2 simple |  |  |  | 361.9 | 50/350/100 | Very High |
| Lung 2 clinical | 68 | 34 | 2 full | 458.4 | 50/500/75 | Very Low |
| Lung 2 complex |  |  |  | 1189.1 | 900/2000/100 | Off |
| Lung 3 simple |  |  |  | 360.8 | 50/350/100 | Very High |
| Lung 3 clinical | 68 | 34 | 2 full | 515.7 | 70/600/75 | Moderate |
| Lung 3 complex |  |  |  | 1200.3 | 1200/2000/100 | Off |

**Supplementary table S4**: Treatment plan information for all gynecological cases. Treatment plans characterized as simple, and complex have been derived from the clinical plan for each case by altering the Monitor Unit (MU) optimization options and the Aperture Shape Controller (ASC) settings.

|  | Prescribed dose (Gy) | | Number of fractions | Arc geometry | Total MU | MU Optimization  Min/Max/Strength | ASC |
| --- | --- | --- | --- | --- | --- | --- | --- |
| Gynecological 1 simple | |  |  |  | 452.2 | 100/450/100 | Very High |
| Gynecological 1 clinical | | 46.8 | 26 | 3 full | 457.9 | 70/600/75 | Very Low |
| Gynecological 1 complex | |  |  |  | 1224.6 | 1200/2000/100 | Off |
| Gynecological 2 simple | |  |  |  | 475.9 | 50/450/100 | Very High |
| Gynecological 2 clinical | | 39 | 13 | 2 full | 736.4 | 50/750/75 | Very Low |
| Gynecological 2 complex | |  |  |  | 1327.8 | 1200/2000/100 | Off |
| Gynecological 3 simple | |  |  |  | 451 | 100/450/100 | Very High |
| Gynecological 3 clinical | | 66/51 | 20 | 3 full | 600.2 | 70/600/75 | Very Low |
| Gynecological 3 complex | |  |  |  | 1252.9 | 1200/2000/100 | Off |

**Supplementary table S5**: Edge Area Metric (EAM), average fraction of edge dose and near-maximum fraction of edge dose (FED_mean_ and FED_2 cm_^3^, respectively), for selected organs at risk (OARs) and the planning target volume (PTV) across all plans. OARs include the rectum (prostate and gynecological cases), contralateral parotid gland (head and neck cases), and spinal cord (lung cases). FED_2 cm_^3^ refers to the highest value of FED received by a 2 cm^3^ volume.

|  | EAM | PTV FED_mean_ (%) | PTV FED_2 cm_^3^ (%) | | OAR FED_mean_ (%) | OAR FED_2 cm_^3^ (%) |
| --- | --- | --- | --- | --- | --- | --- |
| Prostate 1 simple | 0.3 | 23.0 | | 46.3 | 24.5 | 62.5 |
| Prostate 1 clinical | 0.5 | 4.4 | | 22.5 | 17.3 | 55.4 |
| Prostate 1 complex | 0.8 | 40.8 | | 73.0 | 30.7 | 64.3 |
| Prostate 2 simple | 0.5 | 11.6 | | 30.3 | 27.2 | 58.7 |
| Prostate 2 clinical | 0.6 | 32.5 | | 57.9 | 38.7 | 71.3 |
| Prostate 2 complex | 0.7 | 43.9 | | 70.1 | 43.2 | 73.6 |
| Prostate 3 simple | 0.4 | 22.5 | | 46.9 | 35.1 | 73.3 |
| Prostate 3 clinical | 0.7 | 35.4 | | 62.8 | 38.2 | 78.8 |
| Prostate 3 complex | 0.9 | 52.1 | | 76.2 | 38.4 | 79.4 |
| Lung 1 simple | 0.4 | 12.5 | | 36.2 | 24.5 | 47.4 |
| Lung 1 clinical | 0.6 | 30.1 | | 62.8 | 34.6 | 60.8 |
| Lung 1 complex | 0.9 | 43.2 | | 73.7 | 37.9 | 55.5 |
| Lung 2 simple | 0.4 | 7.5 | | 30.5 | 17.9 | 45.9 |
| Lung 2 clinical | 0.5 | 15.2 | | 38.7 | 25.2 | 48.1 |
| Lung 2 complex | 0.9 | 41.7 | | 69.9 | 33.6 | 53.8 |
| Lung 3 simple | 0.4 | 6.7 | | 24.9 | 20.5 | 48.7 |
| Lung 3 clinical | 0.6 | 24.1 | | 48.3 | 28.2 | 56.7 |
| Lung 3 complex | 0.8 | 45.8 | | 69.6 | 39.4 | 64.4 |
| Head & neck 1 simple | 0.5 | 11.4 | | 31.8 | 27.7 | 47.5 |
| Head & neck 1 clinical | 0.6 | 26.0 | | 54.1 | 42.7 | 59.1 |
| Head & neck 1 complex | 0.9 | 51.4 | | 71.2 | 43.5 | 71.8 |
| Head & neck 2 simple | 0.4 | 9.5 | | 33.9 | 17.3 | 37.6 |
| Head & neck 2 clinical | 0.7 | 38.7 | | 67.9 | 38.8 | 62.3 |
| Head & neck 2 complex | 0.9 | 46.8 | | 71.8 | 45.0 | 64.0 |
| Head & neck 3 simple | 0.5 | 12.4 | | 35.7 | 28.0 | 52.3 |
| Head & neck 3 clinical | 0.6 | 27.8 | | 65.5 | 40.3 | 69.8 |
| Head & neck 3 complex | 0.9 | 44.9 | | 76.3 | 42.9 | 67.7 |
| Gynecological 1 simple | 0.2 | 8.5 | | 27.5 | 15.9 | 43.7 |
| Gynecological 1 clinical | 0.4 | 16.3 | | 36.6 | 20.8 | 43.6 |
| Gynecological 1 complex | 0.9 | 37.5 | | 61.0 | 34.5 | 51.9 |
| Gynecological 2 simple | 0.3 | 5.0 | | 24.0 | 22.9 | 65.1 |
| Gynecological 2 clinical | 0.6 | 27.9 | | 46.9 | 38.6 | 66.8 |
| Gynecological 2 complex | 0.8 | 44.7 | | 66.0 | 47.8 | 63.8 |
| Gynecological 3 simple | 0.3 | 10.6 | | 33.5 | 16.1 | 43.5 |
| Gynecological 3 clinical | 0.4 | 16.6 | | 41.5 | 22.0 | 42.8 |
| Gynecological 3 complex | 0.9 | 40.1 | | 67.8 | 38.3 | 54.4 |
